# Altered characteristics of silica nanoparticles in bovine and human serum: the importance of nanomaterial characterization prior to its toxicological evaluation

**DOI:** 10.1186/1743-8977-10-56

**Published:** 2013-11-11

**Authors:** Emilia Izak-Nau, Matthias Voetz, Stefanie Eiden, Albert Duschl, Victor F Puntes

**Affiliations:** 1 Bayer Technology Services GmbH, Leverkusen 51368, Germany; 2 Department of Molecular Biology, University of Salzburg, Salzburg 5020, Austria; 3 Catalan Institute of Nanotechnology (ICN), Campus UAB, Edifici CIN2, Barcelona, Bellaterra 08193, Spain

**Keywords:** Nanocharacterization, Protein corona, Silica nanoparticles, Stability

## Abstract

**Background:**

Many toxicological studies on silica nanoparticles (NPs) have been reported, however, the literature often shows various conclusions concerning the same material. This is mainly due to a lack of sufficient NPs characterization as synthesized as well as *in operando*. Many characteristics of NPs may be affected by the chemistry of their surroundings and the presence of inorganic and biological moieties. Consequently, understanding the behavior of NPs at the time of toxicological assay may play a crucial role in the interpretation of its results.

The present study examines changes in properties of differently functionalized fluorescent 50 nm silica NPs in a variety of environments and assesses their ability to absorb proteins from cell culture medium containing either bovine or human serum.

**Methods:**

The colloidal stability depending on surface functionalization of NPs, their concentration and time of exposure was investigated in water, standard biological buffers, and cell culture media by dynamic light scattering (DLS), zeta potential measurements and transmission electron microscopy (TEM). Interactions of the particles with biological media were investigated by sodium dodecyl sulfate polyacrylamide gel electrophoresis (SDS-PAGE) in bovine and human serum, and extracted proteins were assessed using matrix-assisted laser desorption/ionization-time of flight technique (MALDI-TOF).

**Results:**

It was recognized that all of the studied silica NPs tended to agglomerate after relatively short time in buffers and biological media. The agglomeration depended not only on the NPs functionalization but also on their concentration and the incubation time. Agglomeration was much diminished in a medium containing serum. The protein corona formation depended on time and functionalization of NP, and varied significantly in different types of serum.

**Conclusions:**

Surface charge, ionic strength and biological molecules alter the properties of silica NPs and potentially affect their biological effects. The NPs surface in bovine serum and in human serum varies significantly, and it changes with incubation time. Consequently, the human serum, rather than the animal serum, should be used while conducting *in vitro* or *in vivo* studies concerning humans. Moreover, there is a need to pre-incubate NPs in the serum to control the composition of the bio-nano-composite that would be present in the human body.

## Background

Nanoparticles (NPs) exhibit a variety of unique chemical and physical properties that have made them central components in an array of emerging technologies. Among various NPs that have found commercial application, silica NPs (SiO_2_ NPs) are rapidly becoming a part of our daily life. They are produced on an industrial scale as additives to cosmetics, drugs, printer toners and foods. Functionalized SiO_2_ NPs are being applied in biotechnology and biomedicine as drug delivery systems, in cancer therapy, for enzyme immobilization and for DNA transfection [1-11]. This is in part due to the simplicity of tailoring their surface reactivity via surface functionalization [12,13]. They can also be easily co-synthesized with a variety of fluorophores, in order to produce robust, fluorescent NPs [14].

However, the unique physicochemical properties of SiO_2_ NPs that have made them attractive for the industry may bring potential hazards to human health. Hence, toxicological studies on SiO_2_ NPs have been initiated. SiO_2_ NPs which are available on the market are never pristine particles; they are usually functionalized for their specific application. They can be positively or negatively charged, or have ‘neutral surface’. They can be monodispersed or aggregated. They may be contaminated, what can affect results of their toxicity tests. Consequently, the information about the particles state, in other words, their proper physicochemical characterization prior to their toxicological evaluation seems to be crucial. Moreover, many NPs are likely to undergo significant size distribution or surface chemistry changes while transferred into different environments used for *in vitro* and *in vivo* biological studies [15,16]. Particles may aggregate due to ionic strength of physiological buffers or chemical reactions with molecules derived from the cell culture media [15-20]. Furthermore, the surface properties of the particles can also differ due to adsorption of proteins and reactions of stabilizing groups [21-24]. When NPs enter a biological fluid, proteins and other biomolecules rapidly compete for binding to the NP surface, leading to a formation of a dynamic protein layer that critically defines the biological identity of the particle [25-38]. It is believed that within the first seconds or minutes after immersion of NPs into biological fluids a soft protein corona (PC) is formed and subsequently evolves into a hard PC within hours [39,40]. That may consequently change the NPs properties, affecting biological responses and NPs biodistribution. Thus, the properties of the nano-system, which finally interacts with cells during biological tests, may differ from the initially characterized NPs. Consequently, understanding the NPs behavior at the time of the experiments plays a key role in the interpretation of toxicological results.

In recent years, several studies presenting NPs in different environments with influence on cell viability have been published. It has been shown that different methods of sample preparation had an impact on NPs stability and consequently on the results of toxicity tests [41,42]. In 2004, Rejman et al. have shown that NPs aggregation before uptake altered uptake probability and uptake mechanism and thereby affected biological response [43]. Similarly, it has also been reported that the presence of proteins in a medium affected the entry and intracellular localization of NPs within cells, and thus modulated their potential toxicity [44,45]. Nevertheless, while there is a great deal of studies into biological responses to pristine NPs, for differently functionalized SiO_2_ NPs, there is little information in the literature on their stability in physiological environments and on their interaction with proteins. Indeed, surface functionalized particles are most widely used in the applications of SiO_2_ NPs and are the base of future nanotechnological developments.

The ability of NPs to adsorb proteins has already been shown to depend on the surface coating [34,46]. However, none of the studies until now has presented PC formation for so long time frames, especially on extensively characterized 50 nm SiO_2_ NPs which were varied only in surface chemistry. Even more significant, none of the studies has shown differences in PC formation by comparing serum derived from animal and human.

For the purpose of this study, 50 nm monodispersed fluorescent core/shell SiO_2_ NPs were functionalized with -NH_2_, -SH groups and coated with polyvinylpyrrolidone (PVP), and characterized using a variety of physicochemical methods including zeta potential measurements, dynamic light scattering (DLS), transmission electron microscopy (TEM), scanning electron microscopy (SEM), X-ray photoelectron spectroscopy (XPS), secondary ion mass spectroscopy-time of flight (SIMS-TOF) and X-ray diffraction (XRD). The colloidal stability depending on their surface functionalization, concentration and time was investigated in water, standard biological buffers, and cell culture media. Interactions of the particles with biological media was investigated by sodium dodecyl sulfate polyacrylamide gel electrophoresis (SDS-PAGE) in FBS and human serum, and extracted proteins were assessed using matrix-assisted laser desorption/ionization-time of flight technique (MALDI-TOF).

## Results and discussion

Amorphous 50 nm SiO_2_ NPs encapsulating fluorescein-isothiocyanate (FITC) and functionalized with amino groups (SiO_2__NH_2_), mercapto groups (SiO_2__SH) and polyvinylpyrrolidone (SiO_2__PVP) were synthesized as described previously [47]. The presence of different functional groups immobilized onto the NPs surface was monitored by zeta potential measurements, XPS and SIMS analysis. Full information about the NPs characterization as synthesized is shown elsewhere and the results are summarized in Table 1[47].

**Table 1:** Physico-chemical characterization of nanoparticles

**Name**	**SiO**_**2**_	**SiO**_**2**_**_NH**_**2**_	**SiO**_**2**_**_SH**	**SiO**_**2**_**_PVP**
**Shape**	TEM: spherical			
**Concentration**	2.0% (wt/wt); 1.5x10^14^ NPs mL^-1^			
**Crystal structure**	XRD: amorphous			
**Size/size distribution**	DLS: 58.2±2.6 nm;	DLS: 66.0±3.3 nm;	DLS: 61.3±3.5 nm;	DLS: 67.6±2.6 nm;
	PDI[a] = 0.055	PDI = 0.082	PDI = 0.067	PDI = 0.079
	TEM: d_50_ = 50 nm	TEM: d_50_ = 54 nm	TEM: d_50_ = 56 nm	TEM: d_50_ = 53 nm
	d_90_ = 55 nm	d_90_ = 61 nm	d_90_ = 63 nm	d_90_ = 57 nm
**Surface chemistry**	XPS: Atom%:	XPS: Atom%:	XPS: Atom%:	XPS: Atom%:
	O 62.8, Si 25.6,	O 57.8, Si 24.3,	O 61.8, Si 25.6,	O 44.5, Si 33.5,
	C 11.6	C 16.1, N 1.8	C 12.6, S < 1	C 18.0, N 3.9
	SIMS: Si_x_O_y_,C_6_H_15_O_3_Si	SIMS: Si_x_O_y_,F, (H_2_N(CH_2_)_3_Si(OC_2_H_5_)_3_)	SIMS: Si_x_O_y_, Cl, ((CH_3_O)_3_Si(CH_2_)_3_SH)	SIMS: Si_x_O_y_, F,C_6_H_9_NO
**Surface charge (Z-potential)**	- 41.71 mV±0.82	+ 42.24 mV±1.49	- 47.73 mV±0.91	- 40.87 mV±1.31
	IEP[b]: ~ pH 3.1	IEP: ~ pH 6.4	IEP: ~ pH 1.3	IEP: ~ pH 4.6

[a] polydispersive index; [b] isoelectric point.

All of the SiO_2_ NPs used in this study were well dispersed in ethanol without any agglomeration. Since many NPs characteristics, especially the state of agglomeration, can be affected by chemistry of the surroundings and the presence of both inorganic and biological moieties, understanding of NP behavior in specific environments is crucial. It has been previously shown that many NPs agglomerate in media with a high electrolyte content due to electrostatic screening effects [48], however, the presence of proteins in colloidal suspension stabilize NPs against agglomeration, even in physiological electrolyte concentrations [37,39,49].

**Figure 1:**
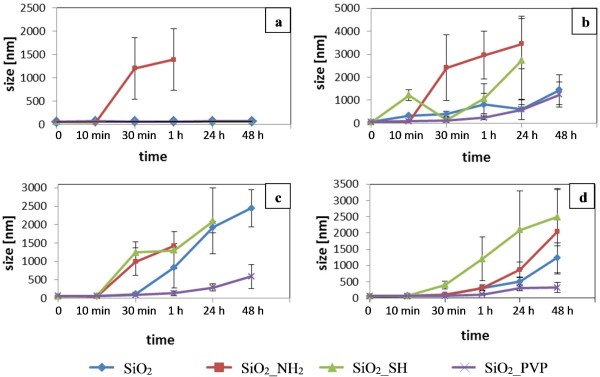
**Time dependent silica nanoparticles stability in different environments.** NPs size was measured by DLS in **(a)** H_2_O, **(b)** PBS, **(c)** DMEM, **(d)** DMEM + 10% FBS; NPs concentration of 1x10^13^ NPs mL^-1^.

According to DLS results, properties of all studied SiO_2_ NPs got slightly altered when transferred from ethanol into Milli-Q (MQ) water (Figure 1-A). However, all of the examined NPs, besides the SiO_2__NH_2_, were stable in MQ water for at least 48 h. Strong aggregation was observed only in case of the amino functionalized NPs already after 10 min. The zeta potential dropped significantly from 42.2 mV to 2.1 mV (see Additional file 1). After 30 min, the agglomerates were big enough to sediment, and their size was more than 1 μm.

In buffers and biological media the NPs are expected to behave differently than in MQ water. In these environments, the ionic strength is usually around 150 mM NaCl, so the electrostatic forces are most likely screened within few nanometers of the surface. For the investigation of such an effect on the SiO_2_ NPs the most common buffer (PBS), and a standard cell culture medium (DMEM), with and without serum, were used. In PBS all of the particles were completely agglomerated/aggregated after 1 h (Figure 1-B). The SiO_2__SH NPs aggregated already after 10 min. In case of the SiO_2__NH_2_ NPs the aggregation was slightly slower, nevertheless after 30 min the size of aggregates was more than 2 μm. The aggregates were generally smaller in case of the non-functionalized SiO_2_ NPs, however, even in this case the aggregation was already detected after 10 min. For the SiO_2__PVP NPs the rate of aggregation was much lower, showing some size increase only after 1 h. Surface charge measurements for all of the SiO_2_ NPs in different environments can be found in Additional file 1.

**Figure 2:**
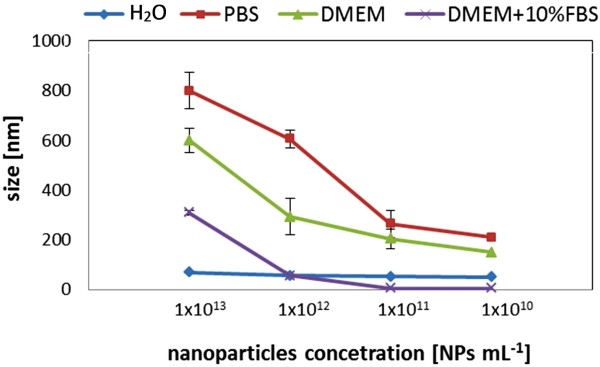
**Concentration dependent silica nanoparticles stability in different environments.** DLS measurements indicated that decreasing concentration of the NPs decreased the rate of their agglomeration.

In DMEM the results were comparable to the ones obtained for PBS (Figure 1-C). The observed similarity was caused by similar ionic strength of these solutions [50]. In DMEM supplemented with 10% FBS serum the aggregation was much diminished (Figure 1-D). However, at the studied concentration of 1×10^13^ NPs mL^-1^ all of the NPs were aggregated after 48 h. In the same time, DLS measurements indicated that decreasing concentration of the NPs decreased the rate of their aggregation (Figure 2). Kretzschmar et al. have also shown that increasing particle concentration of kaolinite resulted in faster growth of aggregates [51]. Burns et al. have found that colloidal polystyrene latex particle aggregation increased with particle concentrations at a fixed level of electrolyte [52]. However, in PBS and DMEM, even at the lowest detectable concentration (1×10^10^ NPs mL^-1^), the NPs aggregated after 1 h of incubation.

In case of DMEM supplemented with 10% FBS, the particles remained stable at a concentration of 1×10^12^ NPs mL^-1^. Measurements at lower NPs concentrations could not be performed due to the higher free proteins content in the samples in comparison to the concentration of the NPs. DLS analysis detected sizes of 5–10 nm, which is the average size of the proteins in FBS. The free proteins could be removed via a centrifugation/washing/sonication procedure. However, it was recognized that in this case the particles size was different than in the case when these procedures were not applied. Too long/fast particles centrifugation, too many washing steps or too long sonication caused the aggregation of the particles. The accurate conditions for silica NPs were found at 15 min centrifugation at 8000 × *g*, one washing step with PBS and gentle NPs pipetting or 5 min in a sonication bath. However, when the sonication procedure was applied, in some cases the size of NPs was lower than the NPs size measured directly in DMEM supplemented with 10% FBS, or even the same like bare particles (without proteins), suggesting that this procedure would partially/totally destroy the formed protein corona.

**Figure 3:**
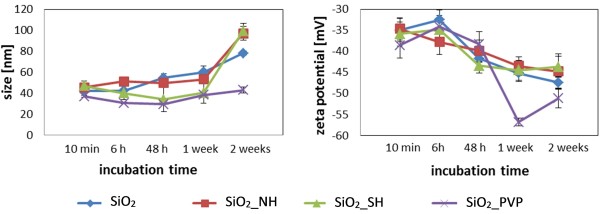
**Time dependent silica nanoparticles stability in biological medium.** DMEM + 10% FBS; NPs concentration of 1x10^12^ NPs mL^-1^; DLS (left panel), zeta potential (right panel).

**Figure 4:**
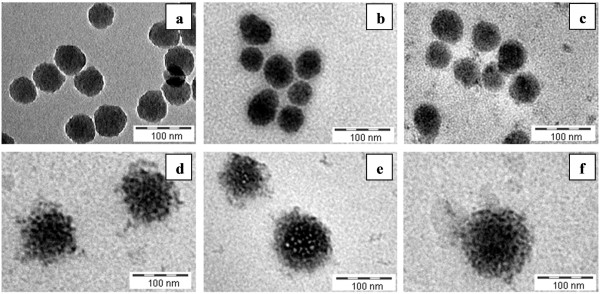
**Time depended silica nanoparticles stability and protein corona formation in biological medium.** NPs concentration of 1x10^12^ NPs mL^-1^, incubation time: **(a)** 0 min, **(b)** 10 min, **(c)** 6 h, **(d)** 48 h, **(e)** 1 week, **(f)** 2 weeks in DMEM + 10% FBS.

Based on the results/concerns presented above, the concentration of 1×10^12^ NPs mL^-1^ was found to be the most suitable to study time dependent stability and changes in PC formation on the differently functionalized SiO_2_ NPs. DLS data showed that all of the examined NPs were stable at concentration of 1×10^12^ NPs mL^-1^ in DMEM supplemented with 10% FBS for at least 2 weeks (Figure 3). After 48 h of incubation in the protein solution the particle sizes seemed to slightly rise. The zeta potential values also became more negative. In this case, it would suggest a higher stability of the nano-system. TEM images of plain SiO_2_ NPs after different incubation times are shown in Figure 4. A protein layer surrounded the NPs was observed after 10 min. After 48 h the NPs size increased to 60 nm, and after 2 weeks to 80 nm. Since no NPs aggregation/sedimentation was detected during this time, this suggested that more than a single layer of proteins was immobilized onto the NPs surface, providing an effective protection against NP-NP interactions leading to aggregation. It is worth noting, that a multilayer of proteins implies protein denaturation [53]. This kind of denaturation is rarely observed and is related to a specific surface charge and hydrophobicity.

It has already been shown by Vroman, in 1962, that adsorption of blood serum proteins onto an inorganic surface was time dependent [54]. Vroman assumed that the proteins with the highest mobility attached firstly, and later were replaced by less mobile biomolecules that had a higher affinity to the surface, in a process that took several hours. Thus, the kinetics of serum protein adsorption onto differently functionalized SiO_2_ NPs were additionally investigated in our work. The majority of *in vitro* and *in vivo* tests concerning NPs are performed using bovine serum, mainly because of its availability and traditional use in many assays, as well as for economic reasons. Since the proteins of bovine serum differ from human proteins, the PC formation and its composition may differ as well. To study differences in proteins associations in different serum, FBS and human serum were applied.

**Figure 5:**
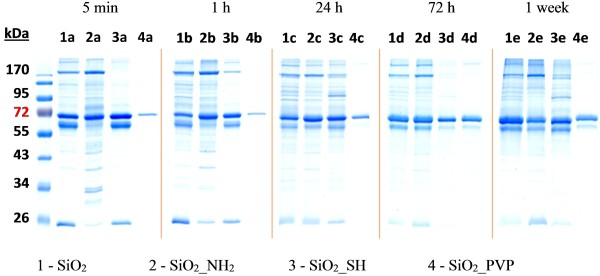
**Bovine proteins immobilized onto nanoparticles surface.** (1) SiO_2_, (2) SiO_2__NH_2_, (3) SiO_2__SH, (4) SiO_2__PVP NPs after (a) 5 min, (b) 1 h, (c) 24 h, (d) 72 h and (e) 1 week of incubation in DMEM supplemented with 10% FBS.

**Figure 6:**
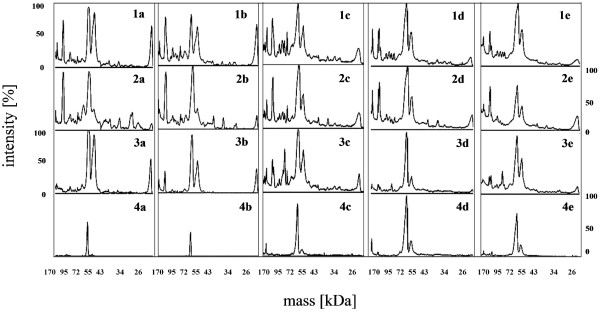
**Amount of bovine proteins immobilized onto nanoparticles surface analyzed with ImageJ software.** (1) SiO_2_, (2) SiO_2__NH_2_, (3) SiO_2__SH, (4) SiO_2__PVP NPs after (a) 5 min, (b) 1 h, (c) 24 h, (d) 72 h and (e) 1 week of incubation in DMEM supplemented with 10% FBS.

SDS-PAGE results indicated that formation of the hard PC took much longer than it was described in the literature before (Figure 5). Previous publications [34,40] have shown that the hard PC was formed after 1 h after NPs incubation in biological medium. In our case, there were still some changes in the hard PC observed even after 24 h. Some slight changes in the proteins adsorption/desorption were even visible after one week. Based on a visual evaluation of the SDS-PAGE gels, the total amounts of adsorbed protein changed not only with time but the extent of protein adsorption/desorption was also functionalization dependent. This might be one of the factors leading to diverse intracellular responses and toxicological outcomes [55]. The amount of proteins was additionally estimated by ImageJ software (Figure 6).

Figure 5 and Figure 6 show that the NPs which attracted the highest amount of FBS proteins were the plain SiO_2_ and the SiO_2__NH_2_. In the case of the plain SiO_2_ NPs, after 72 h incubation time and onwards, there were no significant changes in the PC composition. It is worth noting that, in this case, the amount of the adsorbed proteins increased after 24 h and stayed almost stable for one week, what is in agreement with our TEM results presented in Figure 4. However, some slight changes in the proteins adsorption/desorption were still visible after one week. In the case of the SiO_2__NH_2_ there were small differences observed in the PC between 24 h and 72 h, while after one week the amount of bound proteins was slightly lower. On the SiO_2__SH after 24 h the amount and number of proteins increased and after 72 h decreased again. After one week only slight changes were detectable. The rate of protein adsorption was reduced in the case of the PVP-coated NPs, presumably due to steric repulsion.

**Figure 7:**
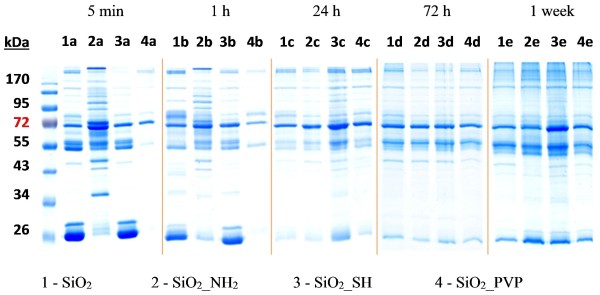
**Human proteins immobilized onto nanoparticles surface.** (1) SiO_2_, (2) SiO_2__NH_2_, (3) SiO_2__SH, (4) SiO_2__PVP NPs after (a) 5 min, (b) 1 h, (c) 24 h, (d) 72 h and (e) 1 week of incubation in DMEM supplemented with 10% human serum.

**Figure 8:**
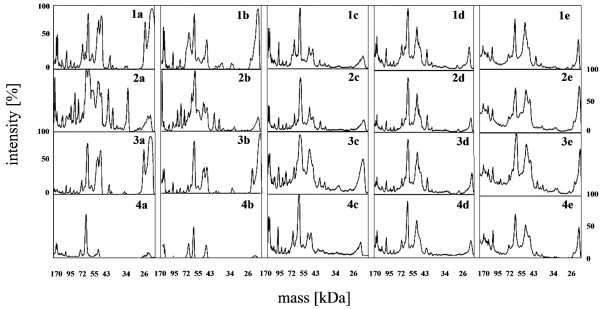
**Amount of human proteins immobilized onto nanoparticles surface analyzed with ImageJ software.** (1) SiO_2_, (2) SiO_2__NH_2_, (3) SiO_2__SH, (4) SiO_2__PVP NPs after (a) 5 min, (b) 1 h, (c) 24 h, (d) 72 h and (e) 1 week of incubation in DMEM supplemented with 10% human serum.

In the case of human serum, the stabilization of the PC was much faster (Figure 7 and Figure 8). After 1 h the PC did not significantly differ from the 5 min incubation. The NPs which attracted the highest number of human proteins were the SiO_2__NH_2._ However, after 24 h the PC still evolved and differences between the different NPs were very small. After 72 h, the same number and amount of proteins were identified on each NP.

In Table 2 we show all of the proteins identified with MALDI-TOF analysis, depending on incubation time and NP functionalization. The analysis indicated that, in the case of FBS, the most abundant protein, irrespective of time or functionalization, was bovine serum albumin (BSA), which is the most prevalent protein in FBS [56]. The other protein detected on every NP regardless of time was apolipoprotein A-I. Apolipoprotein E was detected on the plain SiO_2_ NPs (after 1 h incubation time) and on the SiO_2__NH_2_ (after 24 h). On the plain SiO_2_ alpha 2-macroglobulin precursor was found after 72 h, while it was also adsorbed onto the SiO_2__SH NPs after 24 h. Plasminogen precursor (anticoagulant factor) was also adsorbed on the plain SiO_2_ NPs regardless of time, while on the SiO_2__NH_2_ it disappeared after 1 week, and in the case of the SiO_2__SH it was only detectable after 24 h. Gelsolin isoform b was also detected on the plain SiO_2_ NPs at any time. On the SiO_2__NH_2_ it disappeared after 1 week, and in case of the SiO_2__SH it was detected only after 5 min, 24 h and 1 week. Kelch like protein 9 was adsorbed on the plain SiO_2_ and SiO_2__NH_2_ irrespective of time. It was also present on the surface of the SiO_2__SH after 1 week. Complement C4A was detected on the plain SiO_2_ NPs, the SiO_2__NH_2_ and SiO_2__SH until 24 h of the incubation.

**Table 2:** Bovine and human proteins immobilized onto nanoparticles surface analyzed with MALDI-TOF

	**SiO**_**2**_					**SiO**_**2_**_**NH**_**2**_					**SiO**_**2_**_**SH**					**SiO**_**2_**_**PVP**				
	**1**	**2**	**3**	**4**	**5**	**1**	**2**	**3**	**4**	**5**	**1**	**2**	**3**	**4**	**5**	**1**	**2**	**3**	**4**	**5**
**FBS**																				
Chain A, bovine serum albumin	x	x	x	x	x	x	x	x	x	x	x	x	x	x	x	x	x	x	x	x
Serum albumin precursor	x	x	x			x	x	x					x		x					
Apolipoprotein A-I	x	x	x	x	x	x	x	x	x	x	x	x	x	x	x	x	x	x	x	x
Apolipoprotein E		x	x	x	x			x	x	x			x							
Alpha-2-macroglobulin precursor				x	x								x	x	x					
Plasminogen precursor	x	x	x	x	x	x	x	x	x				x							
Gelsolin isoform b	x	x	x	x	x	x	x	x	x		x		x		x					
Kelch-like protein 9	x	x	x	x	x	x	x	x	x	x					x					
complement C4-A [a]	x	x	x			x	x	x			x	x	x							
**Human serum**																				
Chain A, human serum albumin	x	x	x	x	x	x	x	x	x	x	x	x	x	x	x	x	x	x	x	x
Chain A, lipid-free human apolipoprotein A-I	x	x	x	x	x	x	x	x	x	x	x	x	x	x	x			x	x	x
Apolipoprotein A-IV precursor	x	x				x					x									
Apolipoprotein B-100 precursor	x	x	x	x	x	x	x	x	x	x	x	x	x	x	x	x	x	x	x	x
Apolipoprotein E		x											x	x	x					
Complement C4-A preproprotein						x	x	x	x	x		x	x	x	x					
Complement C4-B-like preproprotein						x	x													
Chain C, human complement component C3c			x	x	x	x	x	x	x	x			x	x	x			x	x	x
Alpha-1-antitrypsin	x	x	x	x	x	x	x	x	x	x	x	x	x	x	x			x	x	x
Chain A [b]	x	x	x	x	x	x	x	x	x	x	x	x	x	x	x	x	x	x	x	x
Coagulation factor II (thrombin)			x	x	x	x	x	x	x	x			x	x	x			x	x	x
Antithrombin III						x	x													
Inter-alpha (globulin) inhibitor H2i						x	x													

(1) 5 min, (2) 1 h, (3) 24 h, (4) 72 h, (5) 1 week of NPs incubation in DMEM supplemented with 10% serum.

[a] predicted; [b] an autoimmune complex between a human IgM rheumatoid factor and IgG1 factor reveals a novel factor epitope and evidence for affinity maturation.

In the case of human serum, the most abundant protein, irrespective of time or functionalization, was human serum albumin (HSA). Other proteins detected on every NP independent of time were apolipoprotein B-100 precursor, and an autoimmune complex between a human IgM rheumatoid factor and IgG 1. Lipid free human apolipoprotein-1 and alpha-1-antitrypsin were detected on every NP irrespective of time, on SiO_2__PVP they appeared after 24 h. Complement C4-B-like preproprotein, antithrombin III and inter-alpha (globulin) inhibitor H2i were adsorbed only on SiO_2__NH_2_ and just at 5 min and 1 h. Human complement component C3c and coagulation factor II (thrombin) were adsorbed on every kind of NP after 24 h. In the case of SiO_2__NH_2_ they were already detectable after 5 min. Apolipoprotein A-IV precursor was only adsorbed at 5 min (SiO_2_, SiO_2__NH_2,_ SiO_2__SH) and 1 h (SiO_2_). Complement C4-A preproprotein was detected on SiO_2__NH_2_ (at each time point) and SiO_2__SH (after 1 h). Apolipoprotein E was attached onto the plain SiO_2_ NPs (only at 1 h) and onto the SiO_2__NH_2_ (at 1 h and 24 h).

BSA and HSA are very similar globular proteins that perform the same functions. However, BSA is composed of 582 amino acid residues while HSA of 585. They also differ in the amino acid composition and charge (BSA: -17, HSA: -15) [57-59]. These differences may affect the conformation, and subsequently regulate proteins adsorption on the NP surface. Consequently the NP interactions with biological surfaces could differ, which may affect biological responses and NPs fate. Besides HSA, the most dominated proteins of human serum immobilized on the NPs surfaces were apolipoproteins. The apolipoproteins participate in lipids transportation in the bloodstream and, as such, are expected to affect the intracellular trafficking and transport of NPs. Apo B-100, present in the case of human serum but not in FBS, functions as a recognition signal for the cellular binding and internalization of low-density lipoproteins (LDL). Other proteins found only in the case of human serum are described below. Alpha 1-antitrypsin is a serum trypsin inhibitor; it protects tissues from enzymes of inflammatory cells. Human complement component C3c is a protein of the immune system; it plays a central role in the activation of the complement system and contributes to innate immunity. Complement C4A and C4B are components of the classical activation pathway of the complement system; they provide a surface for interaction between the antigen-antibody complex and other complement components. Coagulation factor II (thrombin) acts as a serine protease that converts soluble fibrinogen into insoluble strands of fibrin, as well as catalyzes many other coagulation-related reactions. Bearing in mind the well known cases of biological nanoparticles as the LDL and HDL, where single surface-expressed proteins dominate the biological impacts, we believe that the presence of the above described proteins could also have significant biological effect.

The results of our study also indicated that there was a clear dependence of SiO_2_ NPs surface functionalization on protein identity and the NP surface characteristics. Changes in the PCs of the differently functionalized SiO_2_ NPs occurred with time, suggesting that the NP properties could be different at different times of biological experiment. This is an important point that should be taken under consideration for *in vitro* and *in vivo* studies in nanomedicine and nanosafety. The resulting corona created a new NP surface, which may play an important role in its interactions with cell surfaces.

It is interesting to note that a complete stripping of the PC may occur in certain environments (such as a cell phagolysosome as previously suggested [60]) causing restoration of an undecorated surface. Therefore, while NPs pass through the body, there may be serial ‘refreshing’ of the PC after cellular uptake by phagocytic cells. However, in neutral protein rich environment of the cytosol, at long time periods, as the hard corona is quite stable, a subsequent exposure of NPs to a new protein environment may lead to only partial displacement of the original hard corona by new molecules [61,62]. The relative stability of a hard corona, once formed, may suggest pre-incubating NPs intended for human use to allow at least some degree of control over the composition of the bio-nano-composite that will be present in the body. Overall, the final composition of the PC potentially depends on the environments that NP has moved through, rather than only on its current environment [62,63]. In these cases, cells ‘see’ a different object, thus the NP interactions with biological surfaces and receptors could be different which may affect biological responses, NP biodistribution and generally NP fate. The surface of the NP immersed in a medium containing FBS differed from the surface of the NPs in a medium of human serum. Therefore, it seems advisable to verify *in vitro* or *in vivo* studies concerning humans using human serum, rather than relying exclusively on animal serum.

## Conclusions

Surface properties were found to play a significant role in determining NP behavior in different environments. Ionic strength, pH and biological macromolecules completely transformed the NP surface properties and potentially its biological effects. It was recognized that all of the studied SiO_2_ NPs tended to agglomerate/aggregate after relatively short time periods in all buffers and biological media. The aggregation depended not only on the NPs functionalization but also on their concentration and the incubation time. Aggregation was much diminished in a medium containing serum. The PC formation depended on time and NP functionalization, and varied significantly in different types of serum. The resulting corona created a new NP surface, which plays an important role in NP interactions with cell surfaces. Since the PC formation was observed to depend upon which kind of serum was applied, the human serum, rather than the animal serum, should be used while conducting *in vitro* or *in vivo* studies concerning humans.

We suspect that conflicting results of toxicity tests concerning the same nanomaterial (such as SiO_2_ NPs), may to some extent be due to insufficient characterization of the studied materials as well as to varied conditions used during their toxicological evaluation. Different procedures of samples preparation (different buffers/media/serum, different NPs concentration and their incubation time in certain environment) are likely to change the properties of the NPs, as shown above, and give rise to different test results. Hence, researchers must pay closer attention not only to the proper nanomaterial characterization as synthesized, but also to its characteristics under the toxicity tests conditions.

## Methods

### Nanoparticles preparation

Highly concentrated, spherical core/shell 50 nm SiO_2_ NPs encapsulating fluorescein-isothiocyanate (FITC, ≥90%, Fluka, Germany) were synthesized with a modified Stöber method as described previously [47,64]. The NPs surface was additionally coated with polyvinypyrrolidone (PVP, K-15, Sigma-Aldrich, Germany) and modified to generate amino and mercapto functionalities by the addition of organosilanes, such as 3-aminopropyltriethoxysilane (APTES, 98%, Alfa Aesar, Germany) and 3-mercaptopropyltrimethoxysilane (MPTMS, Sigma-Aldrich, Germany) respectively [47,65-67].

### Physicochemical characterization

The particles hydrodynamic size/size distribution and zeta potential were measured by a Zetasizer 3000 HSa, Malvern Instruments. The NPs size was determined by dynamic light scattering (DLS) technique using a He-Ne laser (633 nm) as light source. The stock suspension was diluted to result in a count rate of 100–500 kcps. Particle sizing measurements were performed in 10 mm quartz cuvettes at 25°C. The results were expressed as average values of number, volume or intensity size distribution. The zeta potential was determined by laser Doppler electrophoresis (LDE) using a quartz capillary electrophoresis cell. All of the measurements were performed in triplicate for a single batch of NPs, and the results shown are the average of the three measurements.

The primary NPs size and shape were determined using a Phillips CM20 transmission electron microscope (TEM) working at 200 keV. For TEM analysis, stock NP suspensions were diluted 1:100 and 3 μL were pipetted onto cobalt grids covered with polyvinyl formal/carbon (S162, Plano GmbH) and subsequently left to evaporate. A series of images were selected to estimate particle size/size distribution using the analySiS pro software (Olympus).

The primary NPs size and shape were additionally measured using a FEI Sirion 100 T scanning electron microscopy (SEM) working at 10 keV. For SEM analysis, 20 μL stock suspensions were dried directly on the carbon adhesive pad of a SEM sample holder.

The chemical and elemental composition of NPs were examined with a PHI VersaProbe 5000 scanning X-ray photoelectron spectroscopy (XPS), using a monochromated Al Kα X-ray beam scanned over 600 μm × 400 μm area (200 μm diameter/50 W X-ray beam) or 1400 μm × 100 μm (100 μm diameter/100 W X-ray beam) at a fixed take-off angle of 45°. For XPS analysis, the stock suspensions were dried on an indium surface. Spectra evaluation was performed using MultiPack-Version 9.2 software (Physical Electronics). The results in% were derived from relative concentrations of elements and their chemical bonds from line shape analyses.

The surface chemistry measurements were performed using a time of flight-secondary ion mass spectrometry IV (ToF-SIMS, ION-TOF). The primary ion species used was 10 keV Ga^+^, scanning an area of typically 150x150 μm^2^. For SIMS analysis, the stock suspensions were dried on a silicon surface.

Crystallite size and crystalline phase were evaluated by X-ray diffractometer (XRD) PANalytical EMPYREAN PIXcel with 3D Counter, operating at a voltage of 40 kV and a current of 40 mA with Cu Kα and Kβ radiation. For XRD analysis, the stock suspensions were dried on a silicon surface.

NPs concentration was additionally analyzed with halogen moisture analyzer (HR73, Mettler Toledo). One gram of the stock solution was placed onto an analyzer plate and left for the solvent evaporation to give the wt/wt% value.

### Nanoparticles stability in aqueous/biological environments

To determine the NPs stability in different environments, the NPs dispersions were prepared in sterile Millipore water (MQ), phosphate buffered saline (PBS; pH 7.4), Dulbecco’s modified Eagle’s medium (DMEM, Sigma-Aldrich, USA) and DMEM supplemented with 10% inactivated fetal bovine serum (FBS, Sigma-Aldrich, USA). The NPs samples were incubated at 37°C in a CO_2_ incubator for different periods of time. The NPs were analyzed with DLS, zeta potential and TEM.

### Sodium dodecyl sulfate polyacrylamide gel electrophoresis (SDS-PAGE)

To investigate the NPs interactions with biomolecules, the NPs dispersions (1×10^12^ NPs mL^-1^) were prepared in DMEM supplemented with 10% of inactivated FBS or human serum from human male AB plasma (Sigma-Aldrich, USA). The NPs were incubated for different periods of time at 37°C in a CO_2_ incubator and centrifuged (15 min, 8000 × *g*). The NPs pellets were washed three times with PBS, through gentle pipetting, to remove non-bound proteins. Bound proteins were eluted from the NPs and separated on 12% SDS-PAGE [68] as described before [40]. A protein marker (Fisher BioReagents™ EZ-Run™, Fisher Scientific, USA) was run on every SDS-PAGE. All of the gels were analyzed with ImageJ software [69]. The intensity of the bands was calculated for whole gels (100%-the strongest signal/band, 0%- pure gel/no band). All of the gels were prepared in the same way, making them possible to direct comparison.

### Matrix assisted laser desorption/ionization-time of flight mass spectrometry (MALDI-TOF)

To analyze the proteins bound to the NPs, the proteins bands were dissected from the SDS-PAGE and washed three times with 50 mM ammonium bicarbonate. The samples were treated for 20 min with 20 mM dithiothreitol at 60°C. Afterwards, the samples were left for 15 min in 25 mM Iodoacetamide at 37°C and subsequently digested with 30 ng trypsin/band for 4 h and at 37°C.

The samples were analyzed with MALDI-TOF UltrafleXtreme (Bruker Daltonics) using a positive method operating in reflectron mode and 25 kV acceleration voltage. The analysis was calibrated using external calibrants (Bruker Daltonics).

## Abbreviations

NPs: Nanoparticles; PVP: Polyvinylpyrrolidone; FITC: Fluorescein-isothiocyanate; PC: Protein corona; FBS: Fetal bovine serum; DMEM: Dulbecco’s modified Eagle’s medium; MQ: Millipore water; PBS: Phosphate buffered saline; DLS: Dynamic light scattering; TEM: Transmission electron microscopy; SEM: Scanning electron microscopy; XPS: X-ray photoelectron spectroscopy; TOF-SIMS: Time of flight-secondary ion mass spectroscopy; XRD: X-ray diffraction; SDS-PAGE: Sodium dodecyl sulfate polyacrylamide gel electrophoresis; MALDI-TOF: Matrix-assisted laser desorption/ionization-time of flight.

## Competing interests

The authors declare that they have no competing interests.

## Authors’ contributions

EI, MV, SE, VFP and AD conceived and participated in the design of the study. EI carried out the synthesis and characterization of the NPs, studied the NPs stability in aqueous/biological environments, and investigated interactions of the particles with proteins by SDS-PAGE and subsequently by ImageJ software. All authors were involved in critical review. All authors read and approved the final manuscript.

## Supplementary Material

Additional file 1 Zeta potential values of differently functionalized silica nanoparticles after incubation in various environments at different time. Click here for file
