# Supplementary material for: Altered characteristics of silica nanoparticles in bovine and human serum: the importance of nanomaterial characterization prior to its toxicological evaluation
Source: Part Fibre Toxicol. 2013 Nov 11;10:56. doi: 10.1186/1743-8977-10-56 (PMC3829099; doi:10.1186/1743-8977-10-56)
Supplement: Additional file 1 — Zeta potential values of differently functionalized silica nanoparticles after incubation in various environments at different time. [file 1743-8977-10-56-S1.pdf]

Incubation time	Zeta potential [mV] MiliPore H2O	PBS pH 7.4	DMEM	DMEM+10% FBS
SiO_2 ethanol			-41.7+/-0.8	
10 min	-51.6+/-0.7	-20.4+/-0.5	-19.2+/-0.4	-30.3+/-0.7
30 min	-50.8+/-0.8	-21.0+/-0.3	-17.0+/-1.3	-31.9+/-1.7
1 h	-52.2+/-1.2	-19.7+/-0.8	-18.1+/-0.9	-29.8+/-1.7
24 h	-52.8+/-0.3	-20.5+/-0.6	-20.1+/-0.6	-26.7+/-0.5
48 h	-53.1+/-4.3	-22.3+/-1.0	-17.3+/-1.7	-28.2+/-2.3
SiO_2_NH_2 ethanol			42.2+/-1.5	
10 min	2.1+/-1.1	15.1+/-0.9	10.1+/-0.6	-31.3+/-1.7
30 min	3.8+/-0.2	13.0+/-1.1	12.2+/-2.1	-30.9+/-1.1
1 h	-0.3+/-0.2	2.8+/-0.1	-0.5+/-1.1	-25.5+/-0.7
24 h	-4.2+/-1.1	-15.3+/-2.0	-5.0+/-2.9	-26.5+/-0.5
48 h	-6.2+/-0.3	-15.9+/-0.3	-5.9+/-0.9	-25.2.2+/-1.2
SiO_2_SH ethanol			-47.7+/-0.9	
10 min	-55.2+/-0.6	-18.4+/-1.3	-21.4+/-1.6	-30.3+/-0.8
30 min	-52.3+/-0.2	-19.2+/-0.3	-22.6+/-0.7	-30.9+/-1.0
1 h	-56.7+/-1.2	-19.7+/-0.4	-21.7+/-0.5	-29.4+/-1.1
24 h	-50.0+/-2.1	-17.7+/-1.5	-23.2+/-1.8	-26.4+/-0.5
48 h	-44.5+/5.9	-21.5+/-1.1	-23.5+/-2.1	-28.2+/-1.3
SiO_2_PVP ethanol			-40.9+/-1.3	
10 min	-44.6+/-0.6	-5.7+/-0.6	-13.7+/-0.9	-30.3+/-0.7
30 min	-43.2+/-0.4	-6.8+/-1.3	-16.8+/-1.5	-31.9+/-1.7
1 h	-40.0+/-1.2	-7.8+/-0.5	-10.7+/-0.9	-33.8+/-1.7
24 h	-41.2+/-3.4	-6.9+/-0.4	-10.9+/-0.4	-34.8+/-0.5
48 h	-40.5+/-2.1	-8.6+/-0.6	-12.7+/-0.7	-33.2+/-3.3
